# Telepathology in Low Resource African Settings

**DOI:** 10.3389/fpubh.2019.00264

**Published:** 2019-09-13

**Authors:** Nnamdi Orah, Olorunda Rotimi

**Affiliations:** ^1^Specialist Laboratories Nigeria Limited (The Specialist Laboratories), Lagos, Nigeria; ^2^Leeds Teaching Hospitals NHS Trust, Leeds, United Kingdom; ^3^Department of Cellular and Molecular Pathology, St. James's University Hospital, Leeds, United Kingdom

**Keywords:** external quality, teaching, Africa, low resource, telepathology, digital slides

There is a critical shortage of pathology services in Africa. It is estimated that there are more than 500,000 people per pathologist in much of the continent, with this ratio exceeding 5 million to 1 in some countries (1). In Nigeria, for example, there are about 105 pathologists for an estimated population size of 200 million people, giving a ratio of one pathologist to every circa two million people.

Technological innovation has enabled “leap-frogging” for many developing countries in critical sectors like health. Efforts to bridge the human resource gap in pathology have led to the evolution and spread of diverse telepathology solutions across the continent.

Telepathology is the diagnosis of surgical pathology cases at a distance using real-time video imaging or stored images (2). The definition of the American Telemedicine Association describes telepathology as the following: “A form of communication between medical professionals that includes the transmission of pathology images and associated clinical information for the purpose of various clinical applications including, but not limited to, primary diagnoses, rapid cytology interpretation, intraoperative and second opinion consultations, ancillary study review, archiving, and quality activities (3)”.

Telepathology can be divided into four platforms: static images, whole slide imaging (WSI), dynamic nonrobotic telemicroscopy and dynamic robotic telemicroscopy (4).

Static telepathology involves the examination of precaptured still digital images (snapshots) that can be transmitted via e-mail or stored on a shared server. The benefits derive from its low cost, simple technology required and low maintenance. The images are also small and therefore are easier to manage and store. However, there are several drawbacks including sampling error, limited fields of view, lack of remote controls, and the person taking the images has to have some form of training to select appropriate diagnostic fields. Acquiring images can also be labor intensive.

WSI involves digitization (scanning) of glass slides to produce high-resolution digital slides allowing the consulting pathologist to see the entire specimen at a range of magnifications. It has been shown to be remarkably suitable for telepathology, because digital slides are of high resolution and there is user control of view and magnifications. Its drawbacks are high cost of acquisition and maintenance, the long duration it may take to scan the slides, requirement for large bandwidth internet service and storage difficulties following the large sizes of images produced.

Nonrobotic telemicroscopy involves real-time transmission of live images via a video calling platform, e.g., Zoom®, Facetime®, etc., to consulting pathologists who have no control over the display, while with robotic telemicroscopy the consulting pathologist has control over the live images. Advantages of robotic telepathology include access to the entire slide, user control of the microscope and image with respect to fields and magnification, good image quality, and fast driving speed. Its disadvantages include high cost of the requisite technology, high bandwidth requirements, as well as high costs of acquisition and maintenance (5).

These platforms have provided options for telepathology in developing and underdeveloped countries in sub-Saharan Africa. In this review, we explore the different telepathology solutions that have been adopted in sub-Saharan Africa, their successes and limitations, and potential solutions to these limitations.

## Role of Telepathology in Low Resource Settings

**Figure 1:**
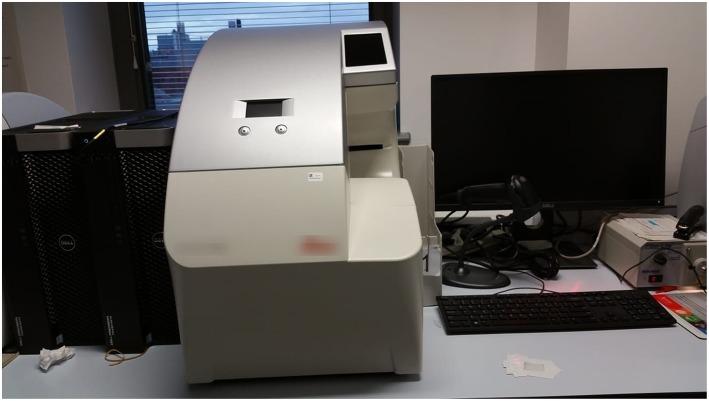
Digital slide scanner.

**Figure 2:**
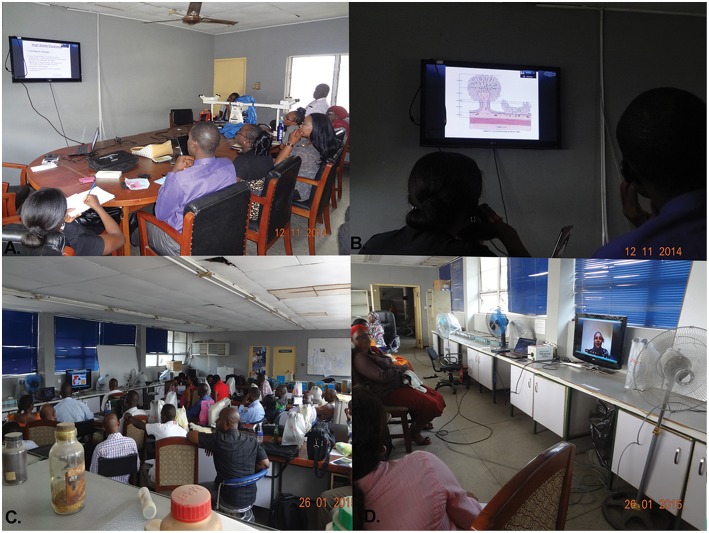
Telepathology in session. **(A)** A cross section of pathology residents in a session at Lagos University Teaching Hospital (Lagos, Nigeria). **(B)** A close-up view of the screen in **(A)**. **(C)** Participants attending the web-based learning sessions at the revision course (Lagos, Nigeria). **(D)** A close-up view of the screen in **(B)**.

1. **Teaching/Education**
  In 2014 and 2015, the Anatomic and Molecular Pathology Department of Lagos University Teaching Hospital, Lagos, Nigeria in collaboration with St. James's Hospital, Leeds, United Kingdom initiated a telepathology postgraduate teaching program using whole-slide imaging (WSI) on the Aperio Digital Pathology System (Leica Biosystems, Buffalo Grove, IL, Figure 1) (6). This collaboration was strictly for teaching purposes only. The scanned slides were accessed via the Leeds virtual pathology website by the participants and real-time discussions were held over Skype® (Microsoft, Redmond, Washington) with the course facilitators in Leeds and Birmingham, United Kingdom. There were 12 sessions of online teaching with an average attendance of 15 participants per session. The participants were later surveyed on the value of this mode of teaching and over 85% of the 42 respondents assessed the lectures as excellent/good as reported by Rotimi et al. (6). Figure 2 shows some pictures taken during these sessions. The advantages of this mode of teaching lie in its convenience and affordability. The participants and the facilitators are able to learn and teach, respectively, stationed in their natural environments. The cost of transportation and other logistics for on-site teaching can also be avoided or greatly minimized.
2. **External Quality Assurance**
  Through the vision and dedicated efforts of a Nigerian pathologist practicing in the UK, Dr. U Ugbokwe, a national (latterly international) diagnostic external quality assurance scheme started in 2010 (see http://www.tslworkshopsng.com/home). For many years, this scheme was slide-based, but in 2016, digital slides (WSI) were introduced. Currently, the selected histology slides for this bi-annual Nigerian and Ghanaian National External Quality Assurance (EQA) scheme are hosted on the Leeds Virtual Pathology server, which has enabled pathologists and pathology residents from Nigeria and Ghana to access the EQA slides at their own convenience. Twenty-five slides are used on each occasion for the EQA. This has eliminated the need to transport the glass slides across states and country borders, thus easing logistic issues. Moreover, the number of participants at the discussion workshops increased from 56 in 2010 to 115 in 2018 with many participants having viewed the digital slides prior to the meetings. It is hoped that the discussions which follow the submission of answers to the EQA will also be held online via teleconferencing facilities soon.
3. **Diagnostics**
  This appears to be the most common use of telepathology in low resource settings. In Malawi, at the Kamuzu Central Hospital, WSI is used to transmit images to collaborating pathologists in the United States for diagnosis (7). The scanned slides are then viewed together with the remote pathologist at a set time. Both parties have equal control over the field of view and magnification, and the final diagnosis is made in agreement with the local pathologist in contrast to models where the foreign pathologist acts as a “stand-alone” consultant. This serves as a good model for teaching as well.
  Similar collaboration between the Department of Pathology at Mulago Hospital in Uganda and Fuerth Hospital in Germany has been reported (8). A total of 96 cases were analyzed. The remote pathologist, using robotic telepatholgy, had control over the focus, magnification and field selection. The slides were viewed over an internet browser-based dynamic imaging system which provided clinical information, gross description, and a digitized microscopy platform. The remote operator had to be trained on the use of this equipment and also on selecting the significant areas for review. It took about 30 min for the pathologist to learn to use the telepathology system and between 4 and 25 min to read a slide remotely.
  This use of telepathology has significantly reduced the turnaround time (TAT) in many underdeveloped countries. This is especially true in countries with a paucity of pathologists. A collaboration between Brigham and Women's Hospital (BWH) in Boston, Massachusetts, USA and Butaro Cancer Centre of Excellence in Rwanda explored the difference in TAT when blocks and slides were sent over to BWH as compared with uploading the slides to the iPath case sharing platform (a static image platform). A total of 3,514 samples were analyzed in total. The study carried out by Muvugabigwi et al. (9) discovered that TAT was significantly shortened for tissue biopsies uploaded to the iPath case sharing platform. Sending the blocks and slides to BWH resulted in a median TAT of 30 days while the iPath platform's median TAT was 14 days. For laboratories dealing with the absence of on-site pathologists, this is a viable and cost-effective alternative to sending blocks/slides abroad for diagnosis.
  The presence of telepathology options for obtaining a second opinion can be a positive factor in the recruitment of pathologists. It has been reported that pathologists have challenges when they work alone (10), and this may affect their retention in remote locations where they are often isolated. However, if static or dynamic forms of telepathology are available, the pathologist can communicate and share diagnosis with other local/foreign pathologists. Pairing remote locations with more experienced tertiary centers is a viable option that can be explored in bridging the gap of pathology service in Africa.
  Apart from diagnostic use, this may also be a form of informal EQA for remote pathologists who may not have the resources or logistics to participate in established local/foreign quality assurance schemes.
  The low operating costs, bi-directional communication, locally responsive services as well as user acceptance have fueled the adoption of telepathology in many underdeveloped countries.

## Challenges

However, telepathology faces many limitations in scaling and widespread adoption. Some of these challenges and proffered solutions include:

1. **Dearth of Infrastructure**
  Power supply and high-speed internet are two major pieces of infrastructure needed for telepathology which are desperately lacking in underdeveloped countries. Rotimi et al. (6) reported participants' difficulties in accessing the virtual pathology website as well as breaks in video communication during the on-line lectures due to poor internet connectivity. Montgomery et al. (7) reported insufficient bandwidth as a limitation to smooth communication and timely loading of slides leading to conference interruptions and rescheduling. Even though a lot of progress has been made in the area of internet penetration across sub Saharan Africa, many remote laboratories remain without basic internet access.
  The government and hospital managers in these countries have a huge role to play in the success of telepathology by making investments in the development of infrastructure and in creating an enabling business environment for telecommunication companies to function. Subsidies and incentives can also be given to telecom companies who invest in rural internet and/or who provide dedicated high-speed internet services for health facilities.
2. **High cost of Telepathology Equipment**
  WSI telepathology is cost-intensive. An Aperio ScanScope system (Leica Biosystems, Buffalo Grove, IL) costs about $85,000 USD exclusive of a $4500 USD service contract (7). Significant capital outlay is also required for the purchase of the necessary bandwidth as well as cloud storage space. This is far beyond the entire budget of most public/private laboratories in underdeveloped countries.
  Cheaper alternatives involving transfer of static slide images have evolved, and successes have been reported in the achievement of concordance between diagnoses made on slide images when compared with diagnoses made on glass slides (9). The drawback to static image transfer as earlier stated includes its lack of control of magnification and focus by the pathologist receiving the images. This may lead to a missed diagnosis with its attendant clinical and medico-legal implications.
  The American Society for Clinical Pathology (ASCP) under its Partners for Cancer Diagnosis and Treatment in Africa has brought together a lot of partners to provide telepathology solutions (11) to underserved populations in Africa. They have donated slide scanners and other digital pathology equipment to laboratories located mainly in East Africa with plans to spread the same over sub-Saharan Africa.
3. **Medico-legal Considerations**
  Legislation regarding the liability of physicians when delivering care across borders is quite unclear (12). African and European legislation have not set forth specific provisions but in the United States, physician licenses are not portable. A draft bill is being considered by the US Congress to facilitate telemedicine endeavors by addressing such legal barriers. There are also legal issues arising from security concerns associated with the confidentiality of medical information on the internet. The solution to this lies in the encryption and anonymization of patient data stored on the internet.
  In our opinion, static and non-robotic telepathology that requires little capital outlay, can make use of facilities already available and requires little maintenance costs will thrive in Africa. This will somewhat ensure the viability of these collaborations and requiring little or no support from donor agencies or international organizations. However, the limitations associated with the use of these particular models of telepathology and the scarcity of resources imply a need for establishment of regional centers of excellence equipped with WSI capabilities where possible.
  In summary, despite the limitations and challenges, telepathology has wide-reaching benefits for healthcare in underdeveloped countries. They serve as avenues for diagnosis, teaching, quality assurance, and research for many pathologists, and they support improvement initiatives hitherto impossible in these countries. The development of collaborations offers significant scientific opportunities for hospitals and academic institutions in underdeveloped countries and those in advanced countries. However, there must be a will to overcome challenges in infrastructure as well as a certain degree of flexibility and resourcefulness in making use of the facilities available.

## Author Contributions

OR and NO contributed equally to the writing and editing of the manuscript.

### Conflict of Interest Statement

NO was employed by ‘The Specialist Laboratories Nigeria Limited'. The remaining author declares that the research was conducted in the absence of any commercial or financial relationships that could be construed as a potential conflict of interest.
